# Enhanced Eryptosis Following Gramicidin Exposure

**DOI:** 10.3390/toxins7051396

**Published:** 2015-04-23

**Authors:** Abaid Malik, Rosi Bissinger, Guoxing Liu, Guilai Liu, Florian Lang

**Affiliations:** Department of Physiology, University of Tübingen, Gmelinstr. 5, 72076 Tuebingen, Germany; E-Mails: malik.abaid@googlemail.com (A.M.); ro.bissinger@gmx.de (R.B.); liugx3692000@gmail.com (G.L.); guilai1986@163.com (G.L.)

**Keywords:** phosphatidylserine, gramicidin, calcium, cell volume, ROS, oxidative stress, eryptosis

## Abstract

The peptide antibiotic and ionophore gramicidin has previously been shown to trigger apoptosis of nucleated cells. In analogy to apoptosis, the suicidal death of erythrocytes or eryptosis involves cell shrinkage and cell membrane scrambling with phosphatidylserine translocation to the erythrocyte surface. Triggers of eryptosis include oxidative stress, increase of cytosolic Ca^2+^ activity ([Ca^2+^]*_i_*), and ceramide. The present study explored, whether gramicidin triggers eryptosis. To this end phosphatidylserine exposure at the cell surface was estimated from annexin V binding, cell volume from forward scatter, red blood cell distribution width (RDW) from electronic particle counting, reactive oxidant species (ROS) from 2',7'-dichlorodihydrofluorescein diacetate (DCFDA) fluorescence, [Ca^2+^]*_i_* from Fluo3- and Fluo4 fluorescence, and ceramide abundance from binding of specific antibodies. As a result, a 24 h exposure of human erythrocytes to gramicidin significantly increased the percentage of annexin-V-binding cells (≥1 µg/mL), forward scatter (≥0.5 µg/mL) and hemolysis. Gramicidin enhanced ROS activity, [Ca^2+^]*_i_* and ceramide abundance at the erythrocyte surface. The stimulation of annexin-V-binding by gramicidin was significantly blunted but not abolished by removal of extracellular Ca^2+^. In conclusion, gramicidin stimulates phospholipid scrambling of the erythrocyte cell membrane, an effect at least partially due to induction of oxidative stress, increase of [Ca^2+^]*_i_* and up-regulation of ceramide abundance. Despite increase of [Ca^2+^]*_i_*, gramicidin increases cell volume and slightly reduces RWD.

## 1. Introduction

Gramicidin, a cationic cyclic peptide antibiotic [1] and ionophore [2,3,4] with contraceptive [5,6,7], antimicrobial [1,5,8] and antiviral [5,9] potency, has previously been shown to trigger apoptosis of nucleated cells [10].

Similar to apoptosis of nucleated cells erythrocytes may enter suicidal cell death or eryptosis, which is characterized by cell shrinkage [11] and phospholipid scrambling of the cell membrane with translocation of phosphatidylserine to the cell surface [12]. Triggers of eryptosis include oxidative stress [12], increase of cytosolic Ca^2+^ activity ([Ca^2+^]*_i_*), ceramide [13], energy depletion [12], activated caspases [12,14,15], activation of casein kinase 1α, Janus-activated kinase JAK3, protein kinase C, p38 kinase and PAK2 kinase [12], as well as inhibition or genetic defects of AMP activated kinase AMPK, cGMP-dependent protein kinase, sorafenib sensitive kinases and sunitinib sensitive kinases [12]. Eryptosis may be elicited by a wide variety of xenobiotics including several peptide antibiotics [12,16,17,18,19,20,21,22,23,24,25,26,27,28,29,30,31,32,33,34,35,36,37,38,39,40,41,42,43,44,45,46,47,48,49,50]. Eryptosis is a model for mitochondria independent suicidal erythrocyte death.

The present study explored, whether and how gramicidin stimulates eryptosis. To this end, human erythrocytes from healthy volunteers were exposed to gramicidin and phosphatidylserine surface abundance, cell volume, reactive oxygen species, [Ca^2+^]*_i_*, and ceramide abundance at the erythrocyte surface quantified utilizing flow cytometry.

## 2. Results and Discussion

The present study explored whether gramicidin elicits eryptosis, the suicidal erythrocyte death characterized by cell shrinkage and cell membrane scrambling with phosphatidylserine translocation from the cell interior to the cell surface.

**Figure 1:**
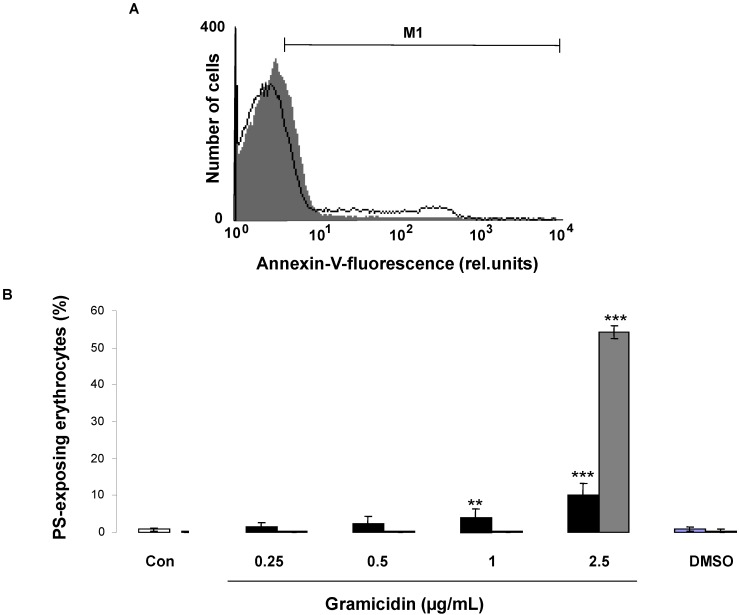
Effect of gramicidin on phosphatidylserine exposure and hemolysis*.* (**A**) Original histogram of annexin-V-binding of erythrocytes following exposure for 24 h to Ringer solution without (grey area) and with (black line) presence of 2.5 µg/mL gramicidin; (**B**) Arithmetic means ± SD of erythrocyte annexin-V-binding (*n* = 12) following incubation for 24 h to Ringer solution without (white bar) or with (black bars) presence of gramicidin (0.25–2.5 µg/mL). As a control, the effect of 1 µL DMSO/mL Ringer is shown. For comparison, the arithmetic mean ± SD (*n =* 4) of the percentage of hemolysis is shown as grey bars. ** (*p <* 0.01), *** (*p <* 0.001) indicate significant difference from the absence of gramicidin (ANOVA).

Erythrocytes exposing phosphatidylserine at their surface were identified according to their annexin-V-binding property, as apparent in flow cytometry. Prior to measurements, the erythrocytes were incubated for 24 h in Ringer solution without or with gramicidin (0.25–2.5 µg/mL) at concentrations previously shown to exert antiviral or spermicidal activity [5]. As illustrated in Figure 1, a 24 h exposure to gramicidin was followed by an increase of the percentage of annexin-V-binding erythrocytes, an effect reaching statistical significance at 1 µg/mL gramicidin concentration. The observations reveal that gramicidin treatment leads to erythrocyte cell membrane scrambling with translocation of phosphatidylserine to the cell surface.

Erythrocyte death could involve hemolysis, a cell death, which is distinct from eryptosis. Hemoglobin concentration in the supernatant was determined in order to estimate the effect of gramicidin on hemolysis. According to the hemoglobin concentration in the supernatant, a 24 h incubation with 0–1 µg/mL gramicidin did not lead to significant hemolysis, but a 24 h incubation with 2.5 µg/mL gramicidin resulted in hemolysis of approximately half of the erythrocyte population.

**Figure 2:**
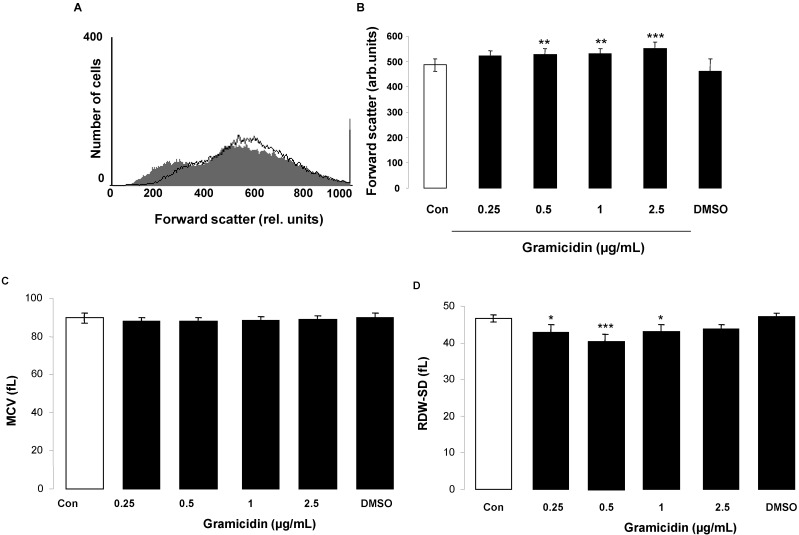
Effect of gramicidin on erythrocyte forward scatter. (**A**) Original histogram of forward scatter of erythrocytes following exposure for 24 h to Ringer solution without (grey area) and with (black line) presence of 2.5 µg/mL gramicidin; (**B**–**D**) Arithmetic means ± SD (*n* = 12) of (**B**) normalized erythrocyte forward scatter (FSC) (**C**) mean corpuscular volume (MCV) and (**D**) red blood cell distribution width (RDW) following incubation for 24 h to Ringer solution without (white bar) or with (black bars) gramicidin (0.25–2.5 µg/mL). For comparison, the effect of 1 µL DMSO/mL Ringer is shown. * (*p <* 0.05), ** (*p <* 0.01), *** (*p <* 0.001) indicate significant difference from the absence of gramicidin (ANOVA).

In order to test, whether gramicidin leads to alterations of cell volume, erythrocyte cell volume was estimated from forward scatter in flow cytometry following a 24 h incubation in Ringer solution without or with gramicidin (0.25–2.5 µg/mL). As shown in Figure 2A,B, erythrocyte forward scatter increased slightly following incubation in Ringer solution with gramicidin, an effect reaching statistical significance at 0.5 µg/mL gramicidin concentration. Gramicidin treatment did not significantly modify mean corpuscular volume determined by electronic particle counting (Figure 2C) and, at lower concentrations (0.25–1 µg/mL) slightly, but significantly decreased the red blood cell distribution width (RDW, Figure 2D), a parameter reflecting heterogeneity of erythrocyte volume [51].

**Figure 3:**
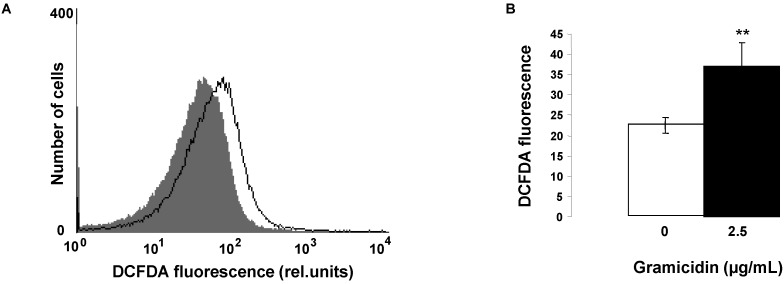
Effect of gramicidin on reactive oxygen species*.* (**A**) Original histogram of 2',7'-dichlorodihydrofluorescein diacetate (DCFDA) fluorescence in erythrocytes following exposure for 24 h to Ringer solution without (grey shadow) and with (black line) presence of 2.5 µg/mL gramicidin; (**B**) Arithmetic means ± SD (*n* = 4) of erythrocyte DCFDA fluorescence following incubation for 24 h to Ringer solution without (white bar) or with (black bar) presence of gramicidin (2.5 µg/mL). ** (*p <* 0.01) indicates significant difference from the absence of gramicidin (*t-*test).

Triggers of eryptosis include oxidative stress. In order to test, whether gramicidin modifies the concentration of reactive oxygen species (ROS), ROS was quantified utilizing 2',7'-dichlorodihydrofluorescein diacetate (DCFDA). As illustrated in Figure 3, a 24 h exposure to gramicidin (2.5 µg/mL) significantly increased the DCFDA fluorescence, an observation pointing to induction of oxidative stress.

**Figure 4:**
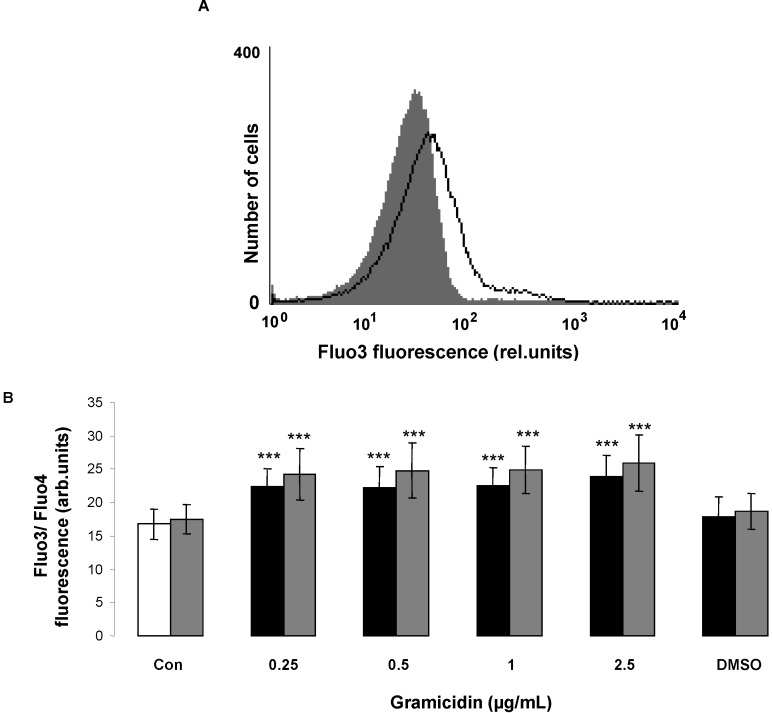

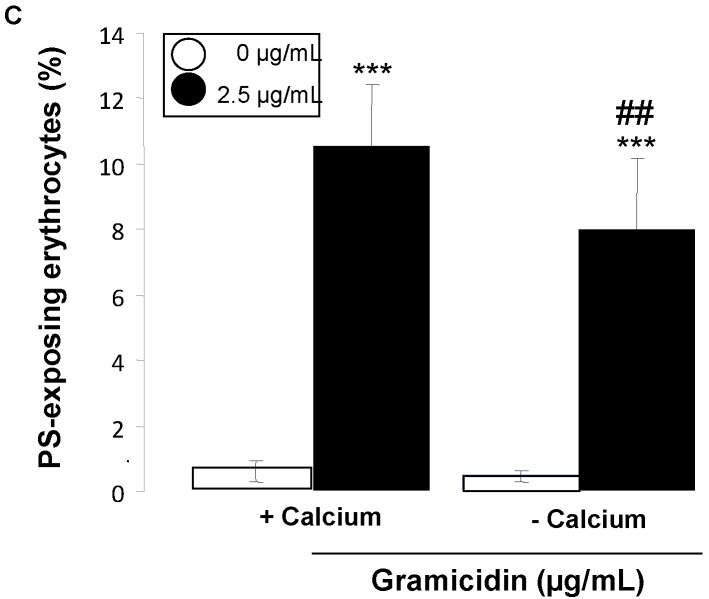
Effect of gramicidin on erythrocyte Ca^2+^ activity and Ca^2+^ dependence of gramicidin- induced phosphatidylserine exposure*.* (**A**) Original histogram of Fluo3 fluorescence in erythrocytes following exposure for 24 h to Ringer solution without (grey area) and with (black line) presence of gramicidin (2.5 µg/mL); (**B**) Arithmetic means ± SD (*n* = 12) of the Fluo3 (arbitrary units, black bars) and Fluo4 (arb. units, grey bars) fluorescence in erythrocytes exposed for 24 h to Ringer solution without (white bar) or with (black bars) gramicidin (0.25–2.5 µg/mL). For comparison, the effect of 1 µL DMSO/mL Ringer is shown (grey bar). *** (*p <* 0.001) indicates significant difference from the absence of gramicidin (ANOVA); (**C**) Arithmetic means ± SD (*n* = 10) of the percentage of annexin V binding erythrocytes after a 24 h treatment with Ringer solution without (white bars) or with (black bars) 2.5 µg/mL gramicidin in the presence (left bars, +Ca^2+^) and absence (right bars, -Ca^2+^) of calcium. *** (*p <* 0.001) indicates significant difference from the respective values in the absence of gramicidin, ## (*p <* 0.01) indicates significant difference from the respective value in the presence of Ca^2+^ (ANOVA).

In order to test, whether gramicidin affected cytosolic Ca^2+^ activity ([Ca^2+^]*_i_*), Fluo3 and Fluo4 fluorescence were measured to quantify [Ca^2+^]*_i_*. As illustrated in Figure 4, a 24 h exposure to gramicidin (0.25–2.5 µg/mL) increased both Fluo3 and Fluo4 fluorescence reflecting increase of cytosolic Ca^2+^ activity ([Ca^2+^]*_i_*), an effect reaching statistical significance at 0.25 µg/mL gramicidin concentration. A further series of experiments explored whether gramicidin-induced cell membrane scrambling was dependent on entry of extracellular Ca^2+^. To this end, erythrocytes were exposed for 24 h to 2.5 µg/mL gramicidin in the presence or nominal absence of extracellular Ca^2+^.

As illustrated in Figure 4C, the effect of gramicidin on annexin-V-binding was significantly blunted in the absence of extracellular Ca^2+^. However, even in the absence of extracellular Ca^2+^ gramicidin significantly increased the percentage of annexin-V-binding erythrocytes. Accordingly, entry of extracellular Ca^2+^ contributed to but did not account for the stimulation of cell membrane scrambling by gramicidin.

**Figure 5:**
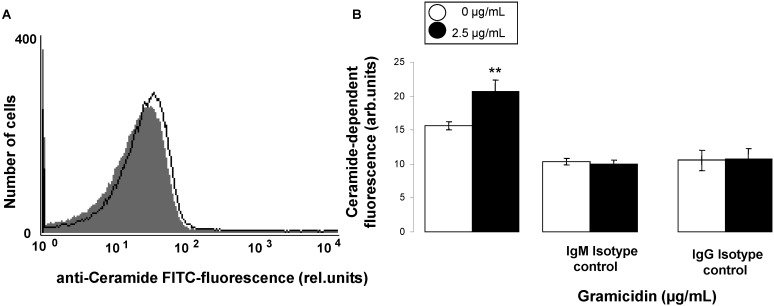
Effect of gramicidin on ceramide abundance. (**A**) Original histogram of ceramide abundance at the erythrocyte surface following exposure for 24 h to Ringer solution without (grey shadow) and with (black line) presence of 2.5 µg/mL gramicidin; (**B**) Arithmetic means ± SD (*n =* 4) of ceramide abundance after a 24 h incubation in Ringer solution without (white bars) or with 2.5 µg/mL gramicidin (black bars). Shown is the respective fluorescence intensity utilizing ceramide antibody (left bars) or isotype IgM (middle bars) or IgG (right bars) antibodies. ** (*p <* 0.01) indicates significant difference from the absence of gramicidin (ANOVA).

As cell membrane scrambling is triggered by ceramide even without Ca^2+^ entry and subsequent increase of [Ca^2+^]*_i_*, the ceramide abundance at the erythrocyte surface was determined utilizing a specific anti-ceramide antibody. As illustrated in Figure 5, a 24 h exposure of erythrocytes to 2.5 µg/mL gramicidin significantly increased the abundance of ceramide at the erythrocyte surface.

**Figure 6:**
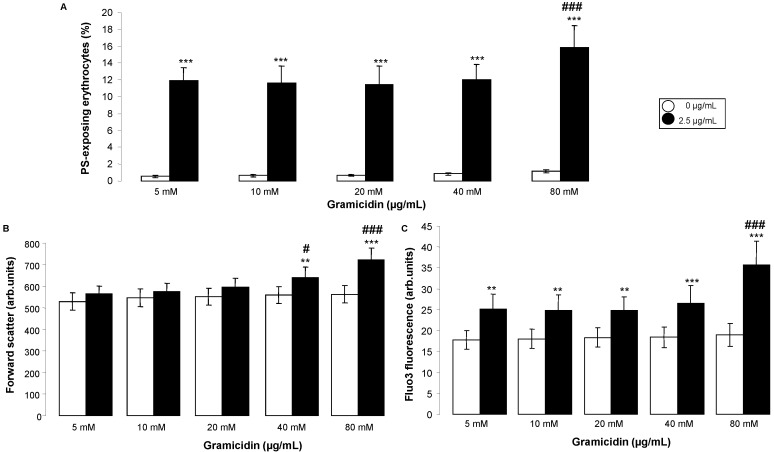
Effect of extracellular K^+^ concentration on gramicidin-induced annexin V-binding, cell swelling and [Ca^2+^]*_i_*. Arithmetic means ± SD (*n =* 9) of the (**A**) percentage of annexin V binding erythrocytes, (**B**) erythrocyte FSC and (**C**) Fluo3 fluorescence after a 24 h treatment in Ringer solution without (white bars) or with (black bars) 2.5 µg/mL gramicidin at 5, 10, 20, 40, and 80 mM extracellular K^+^ concentration. ** (*p <* 0.01), *** (*p <* 0.001) indicates significant difference from the absence of gramicidin (ANOVA). # (*p <* 0.05), ### (*p <* 0.001) indicates significant difference from the respective values at 5 mM K^+^ Ringer.

Additional experiments explored whether the cell membrane scrambling, cell volume and [Ca^2+^]*_i_* was sensitive to replacement of extracellular Na^+^ by K^+^. As illustrated in Figure 6, an increase of extracellular K^+^ concentration from 5 to 20 mM at the expense of extracellular Na^+^ did not significantly influence the effect of gramicidin on annexin V binding, forward scatter or Fluo3 fluorescence. Exposure to 40 mM extracellular K^+^ concentration, however, significantly augmented the effect of gramicidin on FSC, and an increase to 80 mM extracellular K^+^ concentration significantly augmented the gramicidin-induced increase of annexin V binding, FSC and Fluo3 fluorescence.

The present study discloses a novel effect of gramicidin, *i.e.*, the stimulation of erythrocyte cell membrane scrambling with phosphatidylserine translocation to the erythrocyte surface. Cell membrane scrambling is one of the two hallmarks of eryptosis, the suicidal death of erythrocytes. The gramicidin concentration (1 µg/mL) required for stimulation of erythrocyte cell membrane scrambling was within the range of concentrations required for antiviral or spermicidal activity [5]. The concentrations cannot be translated without reservations into *in vivo* concentrations, as in theory protein binding may blunt the effect of gramicidin *in vivo*.

Gramicidin treatment was not followed by erythrocyte shrinkage, the second hallmark of eryptosis. Eryptosis is usually paralleled by cell shrinkage which is at least partially due to increase of cytosolic Ca^2+^ activity ([Ca^2+^]*_i_*) with subsequent activation of Ca^2+^ sensitive K^+^ channels, K^+^ exit, cell membrane hyperpolarization, Cl^−^ exit and thus cellular loss of KCl with osmotically obliged water [11]. According to Fluo3 fluorescence, gramicidin increased [Ca^2+^]*_i_*. Possibly, the cellular loss of K^+^ was overridden by gramicidin mediated entry of Na^+^. Gramicidin is a well-known ionophore permeabilizing the cell membrane for monovalent ions including K^+^ and Na^+^ [4]. Gramicidin slightly decreased RWD indicating that cell volume became more uniform following gramicidin exposure.

K^+^ and Na^+^ gradients across the plasma membrane are dissipated in apoptosis [52], which is usually paralleled by cell shrinkage but may occur without cell volume changes or even with cell swelling [52]. Compelling evidence suggests that monovalent ion transporters contribute to the triggering of cell death independently of their engagement in cell volume regulation [52]. Along those lines apoptosis may be prevented by pharmacological inhibition of Na^+^/K^+^-ATPase [52]. It is tempting to speculate that the dissipation of K^+^ and Na^+^ gradients by ionophores may lead to excessive activity of Na^+^/K^+^-ATPase. However, the proapoptotic mechanisms sensitive to Na^+^/K^+^-ATPase activity remained elusive [52].

The stimulation of cell membrane scrambling by gramicidin may in part be due to oxidative stress, a well-known trigger of suicidal erythrocyte death [12]. Since erythrocytes express oxidant sensitive Ca^2+^ permeable cation channels [12], oxidative stress may contribute to or even account for the gramicidin induced Ca^2+^ entry. The Ca^2+^ entry in turn contributes to the stimulation of gramicidin induced cell membrane scrambling. However, gramicidin triggers cell membrane scrambling even in the absence of extracellular Ca^2+^. Accordingly, the effect of gramicidin on cell membrane scrambling is partially independent from Ca^2+^ entry.

Ca^2+^ independent mechanisms triggering eryptosis include ceramide [12]. According to our observations, the stimulation of cell membrane scrambling is indeed paralleled by an enhancement of ceramide abundance in the erythrocyte cell membrane, which may reflect either formation of ceramide or translocation of ceramide to the erythrocyte surface. In any case, ceramide may well participate in the stimulation of eryptosis [12].

Eryptosis is an important physiological mechanism disposing defective erythrocytes prior to hemolysis [12]. Hemolytic erythrocytes release hemoglobin, which may be filtered in the kidney and precipitate in the acidic lumen of renal tubules thus occluding affected nephrons [53]. Removal of phosphatidylserine exposing erythrocytes may further limit parasitemia and thus favorably influence the clinical course of malaria [54]. Oxidative stress imposed by the malaria pathogen *Plasmodium* activates several ion channels of the infected erythrocyte including Ca^2+^-permeable erythrocyte cation channels [12,55]. Ca^2+^ entry through those channels triggers eryptosis with rapid clearance of the infected erythrocytes from circulating blood [54]. Several genetic erythrocyte disorders including sickle-cell trait, beta-thalassemia-trait, Hb-C and G6PD-deficiency protect against a severe course of malaria at least partially by enhancing the erythrocyte susceptibility to triggers of eryptosis [12,56,57,58]. Enhanced eryptosis may further contribute to the protective effect against malaria of iron deficiency [59], as well as intoxication with several xenobiotics including lead [59], chlorpromazine [60] or NO synthase inhibitors [60]. Whether or not gramicidin may augment oxidative stress and eryptosis of plasmodium infected erythrocytes remains to be shown.

On the other hand, stimulation of eryptosis and subsequent clearance of phosphatidylserine exposing erythrocytes may lead to anemia as soon as the removal of eryptotic erythrocytes surpasses the formation of new erythrocytes [12]. Moreover, phosphatidylserine exposing erythrocytes may bind to endothelial CXCL16/SR-PSO and thus adhere to the vascular wall [61]. Phosphatidylserine exposing erythrocytes may further trigger blood clotting and thrombosis [62,63,64]. Accordingly, phosphatidylserine-exposing erythrocytes may impair microcirculation [13,62,65,66,67,68].

In conclusion, gramicidin triggers erythrocyte cell membrane scrambling, an effect paralleled by and at least partially caused by oxidative stress, increase of cytosolic Ca^2+^ activity and enhanced ceramide abundance at the erythrocyte surface.

## 3. Experimental Section

### 3.1. Erythrocytes, Solutions and Chemicals

Fresh Li-Heparin-anticoagulated blood samples were kindly provided by the blood bank of the University of Tübingen. The study is approved by the ethics committee of the University of Tübingen (184/2003 V). The blood was centrifuged at 120 g for 20 min at 21 °C and the platelets and leukocytes-containing supernatant was disposed. Erythrocytes were incubated *in vitro* at a hematocrit of 0.4% in Ringer solution containing (in mM) 125 NaCl, 5 KCl, 1 MgSO_4_, 32 *N*-2-hydroxyethylpiperazine-*N*-2-ethanesulfonic acid (HEPES), 5 glucose, 1 CaCl_2_; pH 7.4 at 37 °C for 24 h. Where indicated, erythrocytes were exposed to gramicidin (Sigma Aldrich, Hamburg, Germany) at the indicated concentrations, whereby 100 mg gramicidin was solved in 1 mL DMSO. For comparison, the effect of 1 µL DMSO/mL Ringer was tested. In some experiments the extracellular K^+^ concentration was increased up to 80 mM at the expense of Na^+^ (in order to maintain constancy of osmolarity). To achieve changes of 18 mV equilibrium potential for each step, K^+^ concentration was increased from 5 to 10 to 20 to 40 and to 80 mM.

### 3.2. Annexin-V-Binding and Forward Scatter

After incubation under the respective experimental condition, 150 µL suspended cells were washed in Ringer solution containing 5 mM CaCl_2_ and then stained with Annexin-V-FITC (1:200 dilution; ImmunoTools, Friesoythe, Germany) in this solution at 37 °C for 20 min under protection from light. In the following, the forward scatter (FSC) of the cells was determined, and annexin-V fluorescence intensity was measured with an excitation wavelength of 488 nm and an emission wavelength of 530 nm on a FACS Calibur (BD, Heidelberg, Germany). In some experiments erythrocytes were pre-incubated in Ca^2+^ free solution. For determination of annexin-V-binding addition of Ca^2+^ was required during the 15 min incubation with FITC-annexin V. Immediately thereafter the measurement was done so that the exposure to Ca^2+^ was too short to trigger significant phosphatidylserine translocation.

### 3.3. Reactive Oxidant Species (ROS)

Oxidative stress was determined utilizing 2',7'-dichlorodihydrofluorescein diacetate (DCFDA). After incubation, a 100 µL suspension of erythrocytes was washed in Ringer solution and then stained with DCFDA (Sigma, Schnelldorf, Germany) in Ringer solution containing DCFDA at a final concentration of 10 µM. Erythrocytes were incubated at 37 °C for 30 min in the dark and then washed three times in Ringer solution. The DCFDA-loaded erythrocytes were resuspended in 200 µL Ringer solution, and ROS-dependent fluorescence intensity was measured at an excitation wavelength of 488 nm and an emission wavelength of 530 nm on a FACS Calibur (BD).

### 3.4. Intracellular Ca^2+^

After incubation, erythrocytes were washed in Ringer solution and then loaded with Fluo-3/AM (Biotium, Hayward, CA, USA) and Fluo-4/AM (Life Technologies, Carlsbad, CA, USA) in Ringer solution containing 5 mM CaCl_2_ and 5 µM Fluo-3/AM and Fluo-4/AM. The cells were incubated at 37 °C for 30 min and washed twice in Ringer solution containing 5 mM CaCl_2_. The Fluo-3/AM and Fluo-4/AM loaded erythrocytes were re-suspended in 200 µL Ringer. Then, Ca^2+^-dependent fluorescence intensity was measured with an excitation wavelength of 488 nm and an emission wavelength of 530 nm on a FACS Calibur.

### 3.5. Determination of Ceramide Abundance at the Erythrocyte Surface

To determine ceramide abundance, a monoclonal antibody-based assay was used. After incubation, cells were stained for 1 h at 37 °C with 1 μg/mL anti-ceramide antibody (clone MID 15B4; Enzo Life Sciences, Lörrach, Germany) in phosphate-buffered saline (PBS) containing 0.1% bovine serum albumin (BSA) at a dilution of 1:10. After two washing steps with PBS-BSA, cells were stained for 30 min with polyclonal fluorescein-isothiocyanate (FITC)-conjugated goat anti-mouse IgG and IgM specific antibody (Pharmingen, Hamburg, Germany) diluted 1:50 in PBS-BSA. Unbound secondary antibody was removed by repeated washing with PBS-BSA. Isotype control mouse IgM (AbD Serotec, Puchheim, Germany) and mouse IgG (abcam, Cambridge, UK) were used as negative control according to the manufacturer instructions. The samples were then analyzed by flow cytometric analysis at an excitation wavelength of 488 nm and an emission wavelength of 530 nm.

### 3.6. Measurement of Hemolysis

For the determination of hemolysis, the samples were centrifuged (3 min at 1600 rpm, room temperature) after incubation under the respective experimental conditions and the supernatants were harvested. As a measure of hemolysis, the hemoglobin (Hb) concentration of the supernatant was determined photometrically at 405 nm. The absorption of the supernatant of erythrocytes lysed in distilled water was defined as 100% hemolysis. Hemolysis is expressed in % in order to allow comparison with % annexin V binding cells. In one series of experiments, the hemolysis determined as described was compared to the method of Kahn *et al.* [69]. As a result, utilizing the present method the % hemolysis was 1.6 ± 0.3, 1.7 ± 0.3, 1.7 ± 0.3, 1.7 ± 0.4, and 54.1 ± 6.7 at 0, 0.25, 0.5, 1.0 and 2.5 µg/mL gramicidin concentration, respectively. Utilizing the method of Kahn *et al.*, the % hemolysis was 1.6 ± 0.3, 1.3 ± 0.3, 3.4 ± 0.4, 5.1 ± 0.3, and 64.0 ± 7.3 at 0, 0.25, 0.5, 1.0 and 2.5 µg/mL gramicidin concentration, respectively.

### 3.7. Determination of Mean Corpuscular Volume (MCV) and Red Blood Cell Distribution Width (RDW)

For the determination of the mean corpuscular volume (MCV) and the red blood cell distribution width (RDW), the samples were incubated under the respective experimental conditions. After incubation for 24 h, cells were spun down (1600 rpm, 3 min) and the supernatant was discarded. Seventy microliters of the pelleted erythrocytes were analyzed using an electronic hematology particle counter (type MDM 905 from Medical Diagnostics Marx; Butzbach, Germany).

### 3.8. Statistics

Data are expressed as arithmetic means ± SD. As indicated in the figure legends, statistical analysis was made using ANOVA with Tukey’s test as post-test and *t*-test as appropriate; *n* denotes the number of different erythrocyte specimens studied. Since different erythrocyte specimens used in distinct experiments are differently susceptible to triggers of eryptosis, the same erythrocyte specimens have been used for control and experimental conditions.

## 4. Conclusions

Gramicidin triggers erythrocyte cell membrane scrambling, an effect paralleled by and at least partially caused by oxidative stress, increase of cytosolic Ca^2+^ activity and enhanced ceramide abundance at the erythrocyte surface.
